# Letter to the Editor: Bioinformatics Analysis in Mice with Diet-Induced Nonalcoholic Steatohepatitis Treated with Astaxanthin and Vitamin E

**DOI:** 10.3390/ijms18050980

**Published:** 2017-05-04

**Authors:** Chenyu Li, Yan Xu

**Affiliations:** Department of Nephrology, The Affiliated Hospital of Qingdao University, Qingdao 266003, China; licnyu@163.com

## To the Editor,

We read with great interest the article by Kobori and colleagues [1], “Hepatic Transcriptome Profiles of Mice with Diet-Induced Nonalcoholic Steatohepatitis Treated with Astaxanthin and Vitamin E,” which appeared on 30 March 2017 in *International Journal of Molecular Sciences*. Since the results of this article are very interesting for us, we collected original data from GSE93819 (http://www.ncbi.nlm.nih.gov/geo/query/acc.cgi?acc=GSE93819) which has been submitted to NCBI by Kobori et al. and used two different methods to perform the bioinformatics analysis in each group, however, we obtain different results to those in the article: there was no significant difference between the “high-cholesterol, high-cholate, and high-fat diet (CL)” and “CL containing 0.02% astaxanthin (CL + AX)” groups.

We noticed that the author used the MAS5 method which yields a much higher variance in lower-intensity probe sets and which usually causes a high false positive rate in identifying genomic differences [2]. We used RMA, a method with fewer false positives, to preprocess the original data and we utilized the limma (Linear Models for Microarray Analysis) [3] package which is a widely used statistical test to obtain differential expression based on R programming language. The results showed that 111 genes were differentially expressed between the “CL” and “CL + AX” groups (***p* < 0.05 not corrected**, |logFC| > 0.6); however, no gene has a FDR less than 0.05, which means, due to the high false positive rate, that there are no significantly differentially expressed genes between the “CL” and “CL + AX” groups.

In order to verify our results, we used GEO2R [4] (https://www.ncbi.nlm.nih.gov/geo/geo2r/)—a web tool which can identify the differentially expression of genes by comparing samples in a GEO Series—to analyze differentially expressed genes between the two groups again. The results also showed that there was no significant difference between the “CL” and “CL + AX” groups with high FDR.

Additionally, to compare “CL” and “CL+VE”, “CL+ AX” and “CL+ vitamin E”, our results also have a high false positive rate and there was no significant difference between the three groups.
